# Does the Computed Tomography Hounsfield Units Change Predict Response to Perioperative Chemotherapy in Patients with Gastric Adenocarcinoma

**DOI:** 10.7150/jca.67734

**Published:** 2022-02-28

**Authors:** Sener Cihan MD, Suzan Onol MD, Selma Sengiz Erhan MD

**Affiliations:** 1 Department of Medical Oncology, University of Health Sciences, Prof. Dr. Cemil Tascioglu City Hospital, 34384, Istanbul, Turkey.; 2 Department of Radiology, University of Health Sciences, Prof. Dr. Cemil Tascioglu City Hospital, 34384, Istanbul, Turkey.; 3 Department of Pathology, University of Health Sciences, Prof. Dr. Cemil Tascioglu City Hospital, 34384, Istanbul, Turkey.

**Keywords:** Adenocarcinoma, Attenuation in computed tomography, Gastric cancer, Pathologic response, Perioperative chemotherapy

## Abstract

**Purpose:** We aimed to investigate whether Computed Tomography (CT) attenuation change is predictive of poor pathological response in patients with gastric cancer (GC) and gastroesophageal junction (GEJ) adenocarcinoma who received perioperative fluorouracil (FU), leucovorin (LV), oxaliplatin, and docetaxel (FLOT) regimen.

**Methods:** This trial was planned as a retrospective single-center study. In the neoadjuvant setting, patients received a regimen that includes docetaxel (50 mg/m^2^), oxaliplatin (85 mg/m^2^), and LV (200 mg/m^2^) with short-term infusional FU (2600 mg/m^2^ as a 24-hour infusion), on day 1 and administered every two weeks for four cycles. Patients were classified as response rates according to the CAP-TRG system (0-1 response or 2-3 response) after completing four cycles of the FLOT regimen.

**Results:** In total, 108 patients with GC and GEJ adenocarcinoma were included in the study. In a univariate analysis, age, histologic grade, T stage, N stage, and change in attenuation were found to be the statistically significant factors (p = 0.034, p =0.038, p = 0.001, p =0.029, and p = 0.022, respectively). In a multivariate analysis, T4 tumors and a higher change in attenuation were found to be important factors associated with poor pathologic response (p = 0.027 and p = 0.038, respectively).

**Conclusion:** Our results demonstrate that a higher decrease in CT attenuation and T4 tumors is associated with a poor response to perioperative FLOT chemotherapy in patients with GC and GEJ adenocarcinoma.

## Introduction

Gastric cancer (GC) is one of the most common cancers worldwide 1. Although there has been a steady decline in gastric cancer mortality, the prognosis of gastric cancer patients remains poor 2. Endoscopic or surgical resection is curative in approximately 90% of early-stage (T1) tumors, but survival drops dramatically for more advanced tumors (T2-4) or those with regional lymph node involvement 3. Several therapeutic approaches, including perioperative or neoadjuvant chemotherapy (NACT), were established to improve patients with stage II or III GC 4,5,6. Recently, a perioperative FLOT regimen that includes docetaxel, oxaliplatin, and leucovorin with short-term infusional fluorouracil, demonstrated a survival benefit for this approach 7. Pathologic complete response (pCR) following preoperative Ctx indicates a favorable outcome in patients with GC or GEJ adenocarcinoma 8.

In patients receiving neoadjuvant chemotherapy (NCT), stromal and cellular changes may be observed in the microscopic examination of the tumor bed, although it being not specific. Neuroendocrine differentiation, giant cells and mitosis are observed alongside cellular changes, eosinophilic cytoplasm, cytoplasmic vacuolization, nuclear atypia and oncocytic differentiation. Stromal changes include giant cell granulomas, foamy histiocytes, hemosiderin-loaded macrophages, bizarre stromal fibroblasts, cholesterol clefts, vascular changes, dystrophic calcification, acellular mucin pools, and frequently detected fibrosis 9. In a study by Becker K et al., they found more than half of the cancer patients that received NCT (66.7%) developed significant mural fibrosis in the tumor bed 11. There are five classic tumor regression grading (TRG) systems, including the Mandard-TRG system, the Japanese Gastric Cancer Association (JGCA)-TRG system, College of American Pathologists (CAP)-TRG system, China-TRG system, and Becker-TRG system 12,13. College of American Pathologists (CAP)-TRG system is commonly used to assess pathologic response by comparing cancer cellularity in core biopsy before treatment with the resected tumor after treatment. Pathologic complete response and near CR are defined as no viable cancer cells and single cells or rare small groups of cancer cells, respectively 12. The accurate prediction of pathologic response is critical to continue or stop treatment and assess resectability concerning surgery and determine the most appropriate surgical procedure to fit the tumor stage considering the benefits and risks of surgery. In previous studies, re-evaluating tumor size by endoscopic ultrasonography and computed tomography (CT) after neoadjuvant chemotherapy in patients with GC appears to be insufficient in predicting pathological response 14,15. Mide kanseri ve c tile ilişkili bir çok çalışma vardır. Ma Z et al. attempted to predict lymphovascular invasion with preoperative multiphasic dynamic CT in patients with advanced gastric adenocarcinoma 16. Liu S et al. aimed to predict the histopathological features of gastric cancers by applying BT tissue analysis. 17. A study that tracks HU alteration in order to assess the response of gastric cancers to chemotherapy has not been done, to our knowledge.

For this reason, different comparison methods are required to evaluate tumor response. HU is one of those methods.

The Hounsfield unit (HU) is a relative quantitative measurement of radio-density used by radiologists to interpret CT images. The absorption/attenuation coefficient of radiation within a tissue is used during CT reconstruction to produce a grayscale image. The physical density of tissue is proportional to the absorption/attenuation of the X-ray beam. HU can have different values depending on the environment such as -1000 for air, 0 for water and 2000 for dense bone (cochlea). It is being used in the diagnosis and follow-up processes of various benign and malignant pathologies. Several studies have demonstrated that mean baseline HU may identify tumor aggressiveness and tumor response in different tumor types 18,19,20. It is most commonly used to evaluate response of gastrointestinal stromal tumor to imatinib therapy in malignant diseases. In accordance with this purpose, CHOI criteria were created by using HU alterations 21.

Therefore, in this study, we aimed to investigate whether CT attenuation change is predictive of poor pathological response in patients with GC and GEJ adenocarcinoma who received perioperative FLOT regimen.

## Materials and methods

### Study Population and Treatment

This trial was planned as a retrospective single-center study. Medical details were obtained from the archive files of patients who had been treated with NACT between 2018 and 2020 for GC and GEJ adenocarcinoma in the medical oncology clinic of Prof. Dr. Cemil Tascioglu City Hospital. The inclusion criteria were as follows: (1) histologically confirmed primary gastric adenocarcinoma; (2) no prior history of surgery that might adversely affect the blood supply to the tumor; (3) no serious heart and renal insufficiency and other important viscera lesions; (4) performed baseline computed tomography before and after chemotherapy; (5) treated with systemic chemotherapy before surgery. The exclusion criteria were as follows: (1) incomplete clinical data at baseline; (2) poor quality ct images (i.e. motion artifacts); (3) patients who have not completed chemotherapy; (4) having received chemotherapy outside the FLOT regimen. 108 patients who met the criteria above were included in this study. Disease staging was performed according to Tumor, Node, Metastasis staging system 8th. Age and pathological results such as T stage, histological type, lymph node status, grade, data were obtained from the archive files of patients. Patients without a pathology report and archive files were excluded. The histological response was assessed according to the CAP-TRG system. Patients were grouped into T stages, N stages, and radiologically response (complete-near complete response, partial response, and stable disease) and classified as response rates according to the CAP-TRG system (0-1 response or 2-3 response) after completion of the NACT.

In the neoadjuvant setting, all patients had received a regimen that includes docetaxel (50 mg/m^2^), oxaliplatin (85 mg/m^2^), and LV (200 mg/m^2^) with short-term infusional FU (2600 mg/m^2^ as a 24-hour infusion), on day 1 and administered every two weeks (FLOT) for four cycles. All patients were evaluated by CT both at diagnosis and after completion of NACT.

### Computed Tomography and Calculation of HU

Computed tomography was performed at baseline and after four cycles of FLOT chemotherapy in all patients. Two radiologists with more than 8 years' experience with GC evaluated the CT images. Computed tomography examinations were performed with a multi-slice scanner (Philips Ingenuity 128 CT Scan) calibrated according to the manufacturer's specifications. An intravenous contrast agent was used. A slice thickness of 2.5 to 5 mm was used according to the size of the lesion. To determine pre-treatment attenuation values of gastric cancer, measurements were performed using free hand circular regions-of-interest (ROIs) covering the largest quantity of gastric pathologic tissue without any partial volume effect with adjacent structures. For post-chemotherapy attenuation values, measurements were performed using free hand circular ROIs covering the same region of pre-treatment. Additionally for each patients, single free hand ROIs value was used to compare pre- and post-treatment attenuation.

### Statement of Ethics

The study was performed following the declaration of Helsinki. The patients gave written informed consent before the study. Both patient consent and the approval of the University of Health Sciences Turkey, Prof. Dr. Cemil Taşçioğlu City Hospital ethics committee approval were received.

### Statistical Methods

SPSS 15.0 for Windows was used for statistical analysis. Descriptive statistics were given as a number and percentages for categorical variables, average, standard deviation, and minimum and maximum for numeric variables. The numerical variables meet the normal distribution condition, and comparisons of more than two independent groups were made using the Kruskal Wallis test, and did not meet the normal distribution condition comparisons of two independent groups were made using Mann Whitney U test. Comparisons of the ratios in the groups were made using the Chi-Square test. The determinant factors were examined by logistic regression analysis, and a statistical significance level of alpha was accepted as p < 0.05.

## Results

In total, 108 patients with GC and GEJ adenocarcinoma were included in the study. Seventy-three patients were male, and the median age was 61 years. The most tumor localizations were cardia, corpus, antrum, and GEJ, respectively. ECOG PS of all patients was 0-1. Intestinal histology was observed in 92 patients. The percentages of patients with histologic grades I, II, and III were 7.4%, 43.5%, and 49.1%, respectively. The number of patients with T2, T3, and T4 disease was 10 (9.2%), 57 (52.8%), and 41 (38.0%), respectively. Five patients had N0 disease. Median sedimentation, CRP, and LDH levels were 32 mm/h, 10 mg/dl, and 186 IU/L, respectively. Median attenuation at diagnosis and after NACT were 60.5 HU and 49.5 HU, respectively, and the median change in attenuation was 12.5 HU. According to CAP-TRG system, 31 (28.7%) patients displayed a score 0-1 response (group A) and 77 (71.3%) patients displayed a score 2-3 response (group B) (Table 1). In radiologic assessment according to RECIST 1.1, the complete or near-complete response was seen in 19 patients (all of had score 0-1 pathologic response), partial response was seen in 63 patients (12 of had score 0-1 pathologic response and 51 of had scored 2-3 response), and the other 26 patients were reported as stable disease (14 of had score 2 pathologic response and 12 of had score 3 response).

According to the response status, the median age was 62.0 years and 59.0 years in groups A and B, respectively (p = 0.039). The male/female ratio, ECOG PS, tumor localization, histology, sedimentation, lymph node status, attenuation at diagnosis, and after NACT were similar in both groups. The numbers of patients with grade I, II, and III disease were 5, 17, and 9, respectively, in group A and 3,30, and 44, respectively, in group B (p = 0.017). The numbers of patients with T2, T3, and T4 were 8, 19, and 4, respectively, in group A and 2, 38, and 37, respectively, in group B (p < 0.001). Median CRP and LDH levels were lower in group A than group B (p = 0.035 and 0.008, respectively). The median changes in attenuation were 7 HU and 14 HU in groups A and B, respectively (p = 0.016) (Table 1).

In a univariate analysis, age, sex, T stage, N stage, histologic grade, CRP, LDH, attenuation at diagnosis, and change in attenuation were assessed to determine which factors were associated with the CAP-TRG 2-3 score. Age, histologic grade, T stage, N stage, and change in attenuation were found to be the statistically significant factors (p = 0.034, p = 0.038, p = 0.001, p = 0.029, and p = 0.022, respectively; (Table 2).

**Figure 1:**
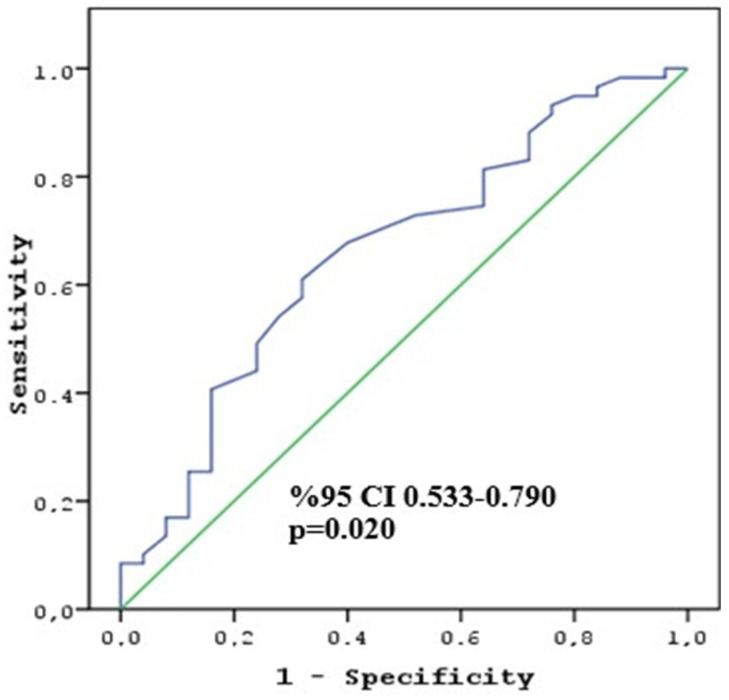
ROC curve of HU change to predict non-responder patients.

In a multivariate analysis, which included variables that revealed statistical significance in the univariate analysis, T4 tumors and a higher change in attenuation were found to be the important factors associated with poor pathologic response (p = 0.027 and p = 0.038, respectively) (Table 3). The cut-off change in attenuation value to determine the CAP TRG 2-3 score was calculated as 10 HU (sensitivity 64.4% and specificity 64.0%) (Figure 1). The second multivariate analysis, including the cut-off value of change in attenuation, revealed that the T4 stage and ≥ 10 HU change were associated with poor response (p = 0.005 and p = 0.014, respectively).

## Discussion

We planned this study retrospectively to detect whether CT attenuation change is predictive of pathological response in patients with GC and GEJ adenocarcinoma who received the perioperative FLOT regimen. In order to rule out the alterations in the efficacy of various chemotherapy regimens, we evaluated only the patients who received the FLOT regimen. We found a serious discrepancy between the restaging by CT and the pathological response, especially in patients with a radiologically partial response. However, we found that higher decrease in attenuation was a negative predictor for poor pathologic response in GC and GEJ adenocarcinoma. In other words, there being no or little to changes in HU rates was coherent with the pathological good response. Additionally, we found that the T4 tumor was associated with a poor pathological response to NACT.

There are various studies focused on factors associated with pathologic response to NACT in GC patients. In several studies, tumor differentiation was found to be significantly associated with pathological response, whereas it was seen that grade did not have a significant effect on pathological response in other studies 22,23,24. Additionally, various studies have demonstrated that a T4 tumor is associated with a poor response to NACT 24,25. In the present study, we found that the T4 stage was associated with a poor response, whereas grade was not related to pathological response, consistent with previous results.

Previous studies have reported that the contrast-enhanced CT demonstrated a greater advantage in evaluating the T3-4 stage of gastric cancer with increased accuracy than the T1-2 stage 26,27. Several studies have focused on the relationship between CT attenuation and prognosis in patients with GC. A study published in 2017 demonstrated that the maximum HU value represents the highest degree of tumor enhancement reflecting the more aggressive tumors 28. In another study that investigated the predictive value of CT at diagnosis for early recurrence and metastasis in GC patients, it was found that compared with patients without recurrence and metastasis, those with recurrence and metastasis demonstrated higher CT values in the arterial and venous phases 29. Both higher T stage accuracy, predictive, and prognostic importance of CT attenuation in GC patients may be related to increased neovascularization 30. However, these studies generally appear to guide initial treatment selection. The perioperative FLOT regimen is widely accepted for initial treatment in patients with ≥ T2-stage or lymph node-positive GC 7. Therefore, evaluation of the response to NACT has become especially important.

In previous studies, re-evaluating tumor downstaging by CT after neoadjuvant chemotherapy in patients with GC appears insufficient in predicting pathological response. In a cohort evaluation within a prospective randomized phase II COMPASS study, the accuracy of radiologic diagnosis after neoadjuvant chemotherapy was assessed, and it was found that overall radiologic accuracy was 42.7% for T-staging and 44% for N-staging. Similarly, in another study, restaging by CT after neoadjuvant chemotherapy in patients with GC was found to be inaccurate 14,15. Furthermore, in the present study, we found a serious discrepancy between the tumor evaluation according to RECIST and the pathological response, especially in patients with a partial radiological response. However, the higher decrease in CT attenuation was an important factor in predicting non-response to NACT in our study. The higher decrease in CT attenuation may be that most of the patients with poor pathological response were radiologically in the partial response group and had T4 tumors. The complete pathological response is associated with marked fibrosis, inflammation, and areas of bleeding to a certain extent, while a partial response is less common with fibrosis 31. Additionally, T3-4 tumors have higher neovascularization and histological grade rather than T1-2 tumors 28. These aggressive tumors have necrotic tissue spontaneously or respond to chemotherapy. Necrosis, bleeding, and inflammation decrease the CT attenuation, whereas fibrosis increases the HU 32.

**Figure 2:**
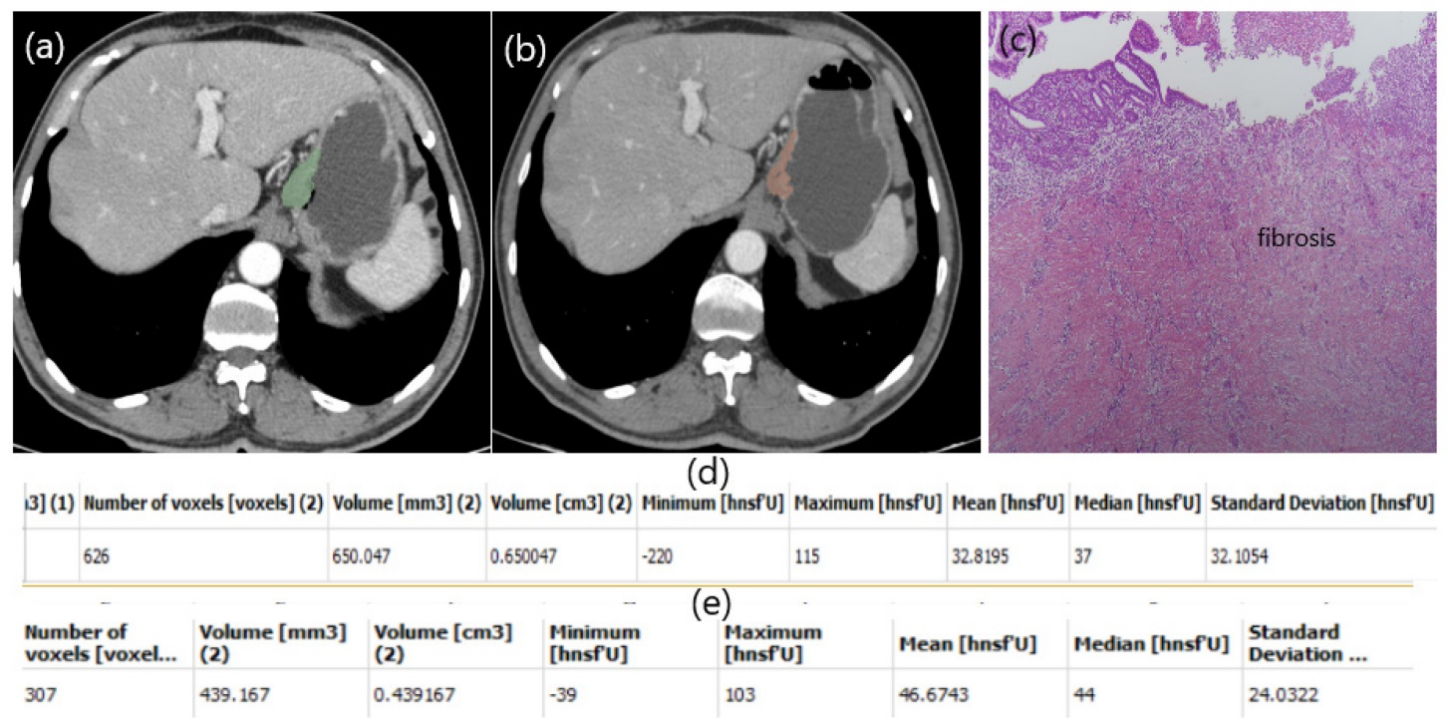
Patient responding well to NAC. Free hand circular ROIs covering the same region of pre (a) and post (b) treatment. HU values pre (d) and post (e) NAC. Diffuse fibrosis in pathology specimen (c).

**Figure 3:**
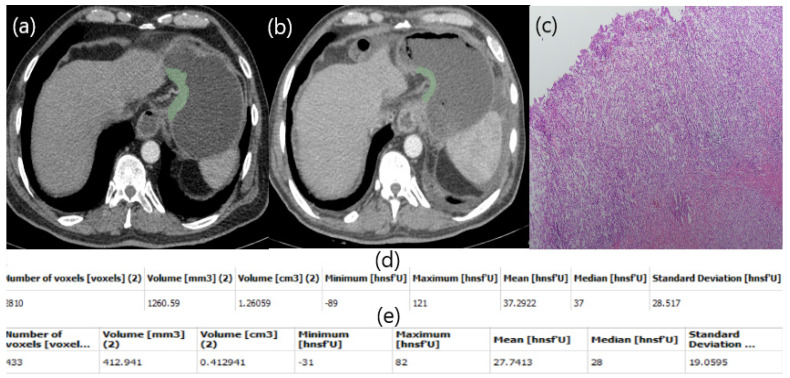
Patient responding poorly to NAC. Free hand circular ROIs covering the same region of pre (a) and post (b) NAC. HU values pre (d) and post (e) NAC. Postop pathology specimen. Almost no fibrosis (c).

In our study, a patient with low HU alteration had pathologically high fibrosis (Figure 2), while another patient with high HU alteration did not respond well to chemotherapy and fibrosis was almost nonexistent (Figure 3). The aim of this study is whether HU alterations can help evaluate the response to chemotherapy in patients with gastric cancer who receiver NAKT. As a matter of fact, at the end of our study the difference in HU alteration being minor was found to be associated with a good pathological response (Figure 2). The relation between HU alteration and histological changes in the tumor bed may be the subject of another study.

Our trial has a few limitations. First, this study was planned retrospectively, which may lead to several biases. Additionally, we could not pre-contrast series for attenuation. Conversely, our study is important because it is the first study focused on this specific subject, has a higher number of patients than other studies, and used a relatively homogeneous group.

Our results demonstrate that a higher decrease in CT attenuation and T4 tumor is associated with a poor response to perioperative FLOT chemotherapy in patients with GC and GEJ adenocarcinoma. Furthermore, our results suggest a more careful evaluation is required in patients with radiologically partial response. However, these results need to be supported by prospective randomized controlled trials.

## Figures and Tables

**Table 1:** Patient's characteristics and comparison of groups

Variables	CAP-TRG score						p
	All patients (n=108)		0-1 (n=31)		>2-3 (n=77)		
	n	%	n	%	n	%	
**Gender**							0.166
Men	73	67.6	24	77.4	49	63.6	
Women	35	32.4	7	22.6	28	36.4	
**ECOG PS**							0.558
0	90	83.3	28	90.3	62	80.5	
1	18	15.7	3	9.7	15	19.5	
**Localization**							0.077
Cardia	30	27.8	8	25.8	22	28.6	
Corpus	25	23.1	12	38.7	13	16.9	
Antrum	29	26.9	8	25.8	21	27.3	
Diffuse	9	8.3	2	6.5	7	9.1	
GEJ	15	13.9	1	3.2	14	18.2	
**Grade**							**0.017**
1	8	7.4	5	16.1	3	3.9	
2	47	43.5	17	54.8	30	39.0	
3	53	49.1	9	29.0	44	57.1	
**Histology**							1.000
Diffuse	16	14.8	4	12.9	12	15.6	
Intestinal	92	85.2	27	87.1	65	84.4	
**T stage**							**<0.001**
T2	10	9.3	8	25.8	2	2.6	
T3	57	52.8	19	61.3	38	49.4	
T4	41	38.0	4	12.9	37	48.1	
**N stage**							0.053
N0	5	4.6	2	6.5	3	3.9	
N1	33	30.6	12	38.7	21	27.3	
N2	38	35.2	14	45.2	24	31.2	
N3	32	29.7	3	9.7	29	37.7	
	**Min/max**	**Median**	**Min/max**	**Median**	**Min/max**	**Median**	**p**
Age	26/78	61	36/76	62	26/78	59	**0.039**
Sedimentation	3/67	32	12/67	30	3/66	34	0.052
CRP	1/54	10	1.84/54	5	1/48	12	**0.035**
LDH	119/892	186	120/350	178	119/892	200	**0.008**
HU at diagnosis	40/120	60.5	40/104	55	40/120	62	0.071
HU after NACT	27/100	49.5	30/94	51	27/100	48	0.574
HU change	-34/52	12.5	-34/41	7	-18/52	14	**0.016**

**Abbreviations:** GEJ: gastroesophageal junction, CRP: C-reactive protein, LDH: Lactate Dehydrogenase HU: Hounsfield units, NACT: neoadjuvant chemotherapy, Min: minimum, Max: maximum, CAP: College of American Pathologist, TRG: Tumor response grade.

**Table 2:** Univariate and multivariate analysis for no response to NACT

Variables		Univariate analysis				Multivariate analysis		
	p	OR	%95 CI		p	OR	%95 CI	
Male vs female	0.170	1.959	0.749	5.124				
**Grade (Ref-1)**	**0.038**							
2	0.178	2.917	0.614	13.846				
3	**0.009**	8.542	1.691	43.139	0.752	0.586	0.021	16.073
**T stage (Ref-T2)**	**0.001**							
T3	**0.013**	8	1.545	41.425	0.206	4.791	0.422	54.343
T4	**<0.001**	37	5.751	238.03	**0.027**	31.718	1.495	672.915
**N stage (Ref N0-1)**	**0.029**							
N2	1.000	1	0.394	2.54				
N3	**0.013**	5.639	1.448	21.952	0.264	5.321	0.283	100.188
**Age**	**0.034**	0.954	0.914	0.997	0.677	1.015	0.946	1.089
CRP	0.243	1.028	0.982	1.076				
LDH	0.929	1	0.996	1.003				
HU at diagnosis	0.106	1.024	0.995	1.054				
HU change	**0.022**	1.042	1.006	1.08	**0.038**	1.11	1.006	1.224

**Abbreviations:** CRP: C-reactive protein, LDH: Lactate Dehydrogenase HU: Hounsfield units.

**Table 3:** Multivariate analysis (Backward method) for no response to NACT

Variables	P	OR	%95 CI	
**T stage (Ref:T2)**	0.014			
T3	0.090	5.594	0.763	41.004
T4	**0.005**	30.601	2.855	328.024
**N stage (Ref:N0-1)**	0.109			
N2	0.127	0.344	0.087	1.353
N3	0.497	1.951	0.284	13.405
HU change <10 vs ≥10	**0.014**	4.607	1.355	15.671

**Abbreviations:** See Table 1.
